# Supplementary material for: Expansion of the fatty acyl reductase gene family shaped pheromone communication in Hymenoptera
Source: eLife. 2019 Feb 4;8:e39231. doi: 10.7554/eLife.39231 (PMC6361591; doi:10.7554/eLife.39231)
Supplement: Supplementary file 2. [file elife-39231-supp2.docx]

**Supplementary File 2**. List of primers and synthetic genes and of generated plasmids and strains.

**Primers:** Restriction sites are underlined.

| **Name** | **Sequence 5'→3'** | **Origin** |
| --- | --- | --- |
| pYEX-BX_seq_F | CATATAGAAGTCATCGA | Clontech |
| pYEX-BX_seq_R | TTTGCAGCTACCACATT |  |
| BlapFAR-A5_RACE_5end-R  BlapFAR-A5_RACE_3end-F  BterBlucFAR-A1_SphI-F | CGCACTATCCCAACTGGCAG  GCATCAAAGCAAAACACCAGTCAG  AAAAAAGCATGCATGAATACGGAATTTACAG | This study |
| BterBlucFAR-A1_NotI-R | AAAAAAGCGGCCGCTTATTAGTACATAACA |  |
| BlucFAR-A1-opt_BamHI-F | AAAGGATCCAACACTGAGTTCACTGAAAAG |  |
| BlucFAR-A1-opt_ EcoRI-R | AAAGAATTCTCAATACATAACTCTCAAAATAATG |  |
| BterFAR-A2_BamHI-F | AAAAAAGGATCCATGAATACGATCAATAGAG |  |
| BlucFAR-A2_BamHI-F | AAAAGGATCCATGGATACGATCAATAGA |  |
| BterBlucFAR-A2_NotI-R | AAAAGCGGCCGCTTATTAGTATACAAAATAT |  |
| BlucFAR-A2-opt_BamHI-F | AAAGGATCCGACACCATTAAC |  |
| BlucFAR-A2-opt_EcoRI-R | AAAGAATTCTCAATAGACGAAATAC |  |
| BterBlucFAR-J-TOPO-F | ATGGTGGAAGTTCTGGTG |  |
| BterBlucFAR-J-TOPO-R | TTACGCAAATTGAGGTATACAATTTC |  |
| BterFAR-J-InFusion-F | TCACCATCACGGATCCGTGGAAGTTCTGGTGGAAC |  |
| BterFAR-J-InFusion-R | GGAAGTTAATGAATTCTTACGCAAATTGAGGTATACA |  |
| BlapFAR-A1_BamHI-F | CGTGGATCCAATACAAAACTTAATGAAAACGAGATAAATG |  |
| BlapFAR-A1_NotI-R | TTTAGCGGCCGCTTACGGCGCCGCATTTATAGATTTAG |  |
| BlapFAR-A1-syn_BamHI-F | AAGGATCCAATACAAAACTTAATG |  |
| BlapFAR-A1-syn_NotI-R | AATCAAGCGGCCGCTTACGGC |  |
| BlapFAR-J_SphI-F | ACAATGCATGCGTGGAAGTTCTGGTGGAA |  |
| BlapFAR-J_NotI-R | ACAAGAATGCGGCCGCTTACGCAAATTGAGGTATACAA |  |
| BlapFAR-A4_BamHI-F | CTTGGATCCGATACAATCAATAAAGAAAG |  |
| BlapFAR-A4_EcoRI-R | CATGAATTCTCAATAAACGATACAGTATAC |  |
| BlapFAR-A5_BamHI-F | GACAAGGATCCGATACAACCGATAAA |  |
| BlapFAR-A5_NotI-R | TAACTGCGGCCGCTCAGTACACAAAATA |  |
| BterBlucFAR-A1_qPCR-F | ACACGGCAATGGTCCTTCAA |  |
| BterBlucFAR-A1_qPCR-R | AAAAATAAATTTCTTTATTCCTATGACGCA |  |
| BterBlucFAR-A2_qPCR-F | AGCAGAGCAAATTGTAGCAAGC |  |
| BterBlucFAR-A2_qPCR-R | ACTACATCCACTCTTCCATCTCTCC |  |
| BterBlucBlapFAR-J_qPCR-F | TGCGAGGATGGATCGACAAC |  |
| BterBlucBlapFAR-J_qPCR-R | TCTTTGGTAGTTGCAGATTTTCGAC |  |
| BlapFAR-A1_qPCR-F | AAGAAAAGGTCTACACCACGAATC |  |
| BlapFAR-A1_qPCR-R | TCGCAACAGTCAATCCTTTG |  |
| BlapFAR1-A1-short_qPCR-F | AATCTATCAGACATCGAAGAATTGATTA |  |
| BlapFAR1-A1-short_qPCR-R | CAGGGAACGGTTCTTTTAGC |  |
| BlapFAR-A4_qPCR-F | AGTGACAGCATATTTCGCTCTG |  |
| BlapFAR-A4_qPCR-R | AAACGATACAGTATACAATCAGTAGTG |  |
| BlapFAR-A5_qPCR-F | AGTTGAAAAATGCCGTGTTGAAG |  |
| BlapFAR-A5_qPCR-R | GTGCGTGGTGAAATATGTTACCGA |  |
| BlapEEF1A_qPCR-F | AGAATGGACAAACCCGCGAG |  |
| BlapEEF1A_qPCR-R | CACAAATGCCACCGCAACAG |  |
| BlapPLA2_qPCR-F | GGTCACACCGAAACCAAATT |  |
| BlapPLA2_qPCR-R | TCGCAACATTTCGTCATTTC |  |
| BterBlucEEF1A_qPCR-F | AGAATGGACAAACCCGTGAG | Horňáková et al., 2010 |
| BterBlucEEF1A_qPCR-R | CACAAATGCTACCGCAACAG |  |
| BterBlucPLA2_qPCR-F | GGTCACACCGAAACCAGATT |  |
| BterBlucPLA2_qPCR-R | TCGCAACACTTCGTCATTTC |  |

**Synthetic genes:** Restriction sites are underlined.

*Bluc*FAR-A1-opt (codon-optimized for yeast) GenBank accession number **MG450697**

GGATCCAACACTGAGTTCACTGAAAAGTCTAACAAGGTCAACTCCATCGAAGGTTTCTACGCTGGTACAGGTATCTTTATCACAGGTGCCTCAGGTTTTGTCGGTAAAGGTTTGTTGGAAAAGTTGATCAGAGTTTGTCCTAGAATCGTTGTATTATTCATCTTGGTTAGACCAAAGAAACATCAAACAATGGAACAAAGATACAAGGAAATCATGGATGACCCTATCTTTGATGACATCAAAGCTAAGAATCCATCCGCATTGAAAAAGGTCCATCCTGTTGAAGGTGACATTTCCTTACCAAAGTTGGGTTTGAGTCAAGAAGATAGAAACATGTTGATAGAAAACGTCAACATCTTGTTTCACGTTGCTGCATCTTTGAACTTCAAGGAACCATTGAACGCCGCTGTAAATACTAACGTCAAGGGTACATTTTCTATAATCGAATTGTGTAACGAATTGAAGCATGTTATATCAGCTGTACACGTCTCTACAGCATATTCAAATGCCAACTTGCCTGAAATAGAAGAAAAGGTTTACTCCACTATCTTACAACCATCTTCAGTAATTGAAACATGCGACAGTTTGGATAAGGAATTGATTAAGTTGTTGGAAGAAAGAATTTTGAAAATACATCCTAACACGTACACCTTCACGAAGAATTTGGCAGAACAAATCTTGTCCAGTTCTTCAACTAACTTCCCAATAGCAATCGTTAGACCTTCTATCATTTCCGCCAGTTTAAAAGAACCATGTCCTGGTTGGTTGGGTAATATTACAGCCCACATAGCTTTGGGTTTGTTTATTTCAAGAGGTTTCGCCAAGATCACCTTAGCTAACCCTGACACTATCACAGATACCGTACCATTAGACTATGTCGTTGATACAATTTTGTGTGCAGCCTGGCATGTTACCTTGCACAGAGATATGAACGTTAAGGTATACAACTGCACCAATAACGCCAGACCAATTAATTACGGTGAATTGAAGGACACTTTTGTCAAGTACGCTATTCAAATACCTATGGATGGTTTAGTTTGGTATCCATGTTGCGCAATGGTTTCTAACAGATACGTATACTCAATCTTGACCTTATTCTTGCATACTTTGCCTGCTTTTATTATGGATATTTTCTTAAGATTGCAAGGTTCAAAGCCAAGAATGATGAAGATCTCTAAGTACTACGATACAATGTCAATCGTTACCAACTACTTCTCCACTAGACAATGGAGTTTCAAAAAGGATAACGTTATTAATATGATGAAAGAAGTCAAGACTTTGGAAGATTCTGACATCGTTAGATTAGATTTGCAAGATATGGACTGGGATAAGTACATCGCTATATGCGTTATCGGTATCAAAAAGTTTATTTTCAAAGAAGACCCAAAGTCCTTAGATGCTGCATTGAGAAGATTGAGTATCTTTTACTGGATTCATCAAATGACTAAAGCCTTCGCTATTATTATCTTATTGACCATTATTTTGAGAGTTATGTATTGAGAATTC

*Bluc*FAR-A2-opt (codon-optimized for yeast) GenBank accession number **MG450704**

GGATCCGACACCATTAACAGAGAAAAGAACGAAAACGCCATTAACAAGGGTTTGAACAAGTTGAATACATTAGAAGAATTTTACGTCGGTAGTGGTATTTTGTTAACTGGTGCAACAGGTTTTGTTGGTAAAGCTGTTTTGGAAAAGTTGATCAGAATGTGTCCAAGAATTGCTGCAATTTTCTTGTTGTTTAGACCAAAGACTGATGAAACAATCGAACAAAGATTCAAGAAATTGATTGATGATCCAATCTATGATGATATCAAGGCAAAGCATCCATCAACTTTGTCTAGAGTTTATCCAATGAGAGGTGACTTGTCATTGCCAGATTTGGGTTTGTCTAGAGAAGATAGAAATTTGTTGTTGGAAAAGGTTAACATCGTTTTCCATGCTGCAGCTACTGTTATGTTTAATGAACCATTGCAAGTTACAATTAATGTTAACACTAAAGGTACAGCTAGAGTTATTGATTTGTGGAACGAATTGAAGCATCCAATCTCATTCGTTCATGTTTCAACTGCATTTTCTAACGCTAACATCCATGAAATCGGTGAAAGAGTTTACACTACATCATTGAAACCATCTGAAGTTATTGATATTTGTAATAAGTTTGATAAAACATCTATTAATCAAATCGAAAAGAAAATTTTGAAAACTTATCCAAATATCTATACTTTTTCTAAAAATTTGGCAGAACAAATCGTTGCTTCTAACTGTAAGGATATGCCAGTTGCTATTGTTAGACCATCAGTTATTGGTCCATCTATGGAAGAACCATGTCCAGGTTGGATTCAAAACATCTCTGCAATCACTGGTATCATGGTTTTGATCGGTAGAGGTTGTGCTACAGGTATTAGAGGTAGAAGAGATGGTAGAGTTGATGTTGTTCCATTGGATTACGTTGTTGATATGATCATCTGTACTGCATGGCATGTTACATTGCATCCAAAGCATGAAGTTAAGGTTTACAACTGTACATCTTCAGCTAACCCAATTAGATGGGGTCAAATGCAACAATTGGTTTTGAAGCATTCAAGAGAAACTCCATTAAACGATACATTGTGGTATCCAAGATGTCCAATCATCGCAAATAAGTACATTTTCAATGTTTTATGTGTTATCCCATACGTTTTGCCAGCTTTTATTATCGATATTTTCTTGAGATTGAGAGGTTCTAAGCCAATCATGATGAAGTTGTTGAAGTTCGGTTACAAGTTGTCTACTTCAGTTTCTCATTTCACTATGAACGAATGGACATTCCAAAGAGATAACTGTTCAGATTTGGCATCTAAGGTTAAGATGTTGCATGATTCAGATATGGTTAAATTAGATTTGAGAGATATGAAGTGGGAAAAGTACATCGTTATATATTTGATGGGTATTAGAAAGTTTATTTTGAAACAAGAATTTCAACCAACAGCTAGACAAAGATTGTCTAGATTGTACTGGATTCATCAAATCACTAAGATTTCAGGTATCATCAGTTTGTTATGGATTATTTTGTATTTCGTCTATTGAGAATTC

*Blap*FAR-A1 GenBank accession number **MG450698**

GGATCCAATACAAAACTTAATGAAAACGAGATAAATGAAAAATTACGTAATGTGAATTCCATTGGGGGATTCTACGCCGGAACTGGAATTCTTATTACTGGTGGGACAGGTTTCGTGGGCAAAGGACTCCTCGAAAAACTGATACGCACGTGTTCACACATCGCTGCTATTTTTATATTGATCCGTCCGAAACGTAACCAAACGATAGAACAACGATTTAAGAAGATAATAGATGATCCGATTTTCGATGGTGTCAGAGCACAAAACCCAGCAATTTTCTATAAAATTCATCTCGTGGAGGGCGACGTGACTCTACCAGATTTAGGTCTTTTGCAAAAAGACAGAGATATGTTGATAGAGAATGTAAACATAGTGTTCCACATTGCGGCCACTATAAATTTCCATCAACCATTGGATATGATTGTCAATGTAAATGTGAAAGGTACCGCTAATATTATCAAACTGTGCAAGGAACTCAAGCATGTAATTAGCGTTGTCTATGTGAGCACAGCTTACAGTAATCCGAATCTATCAGACATCGAAGAAAAGGTCTACACCACGAATCTAGATCCCTCTCTCGTGATGGATATATGCGACCGACAAGACAAAGAATTGATTAATCTGATCGAAGAAAGAATTTTAAAAACGCATCCGAACACATACACGTTCACCAAGAATCTTGCAGAGCAGACAATATCCAACAATAGCAAAGGATTGACTGTTGCGATAGTGCGACCAAGTATAATTTCTTCCTCGCTAAAAGAACCGTTCCCTGGTTGGTTGGTATCTTTTGCTGGACAATCAGGTATCTTCAAGAATATCGGCAATGGTATGGCAAAAGTACTATTGGGTAGGGGAGATGTAATATCAGATATAGTGCCTGTTGATTATGTAGTCGACGCGATAATGTGTGCCGCGTGGCACGTCACGCTACAAATTGATAATAATGTCAAAGTTTACAACTGTACGAGCAGCGCACGTCCCATCAAATTGGGTGAAATCGTAAATATCTTCGTGGAATGTAGCAGAGAAATACCGATGAAAAATACGTTGTGGTATCCGAGTTGTACGATAGTAGCAAACAGATTTGTTTACAATGTACTGAATATACTTCTAAATGTTTTACCTGCGTTTGCCGTGGATATCTTTTTAAGGCTTCGAGGTGGTAAACCAATGGCAATGAATATGAACAAATATTACAATAAATTGGTCGTAGCGACAAGCTACTTCAACTCGAATGAATGGTCCTTCAAAAGAGATAACATTGCCGATATGATAAACAAGGTGAATACCTTGGAAGATGGAAATATTGTTAAACTGGACTTGCAGGATATGGTTTGGAGGAAATATATAGCAAATTACTTGGCGGGAATTAAGAAATTTATTCTGAAAGAAGACCCTAAATCTATAAATGCGGCGCCGTAAGCGGCCGC

**Plasmids:**

| **Name** | **Description** | **Origin** |
| --- | --- | --- |
| pGS1 | pUC57-Simple::*Bluc*FAR-A1-opt | GenScript HK |
| pGS2 | pUC57-Simple::*Bluc*FAR-A2-opt | GenScript HK |
| pGS3 | pUC57-Simple::*Blap*FAR-A1 | GenScript HK |
| pY1 | pYEXTHS-BN::*Bluc*FAR-A1 | This study |
| pY2 | pYEXTHS-BN::*Bluc*FAR-A1-opt |  |
| pY3 | pYEXTHS-BN::*Bluc*FAR-A2 |  |
| pY4 | pYEXTHS-BN::*Bluc*FAR-A2-opt |  |
| pY5 | pYEXTHS-BN::*Bter*FAR-A1 |  |
| pY6 | pYEXTHS-BN::*Bter*FAR-A2 |  |
| pY7 | pYEXTHS-BN::*Bter*FAR-J |  |
| pY8 | pYEXTHS-BN::*Blap*FAR-A1 |  |
| pY9 | pYEXTHS-BN::*Blap*FAR-A1-short |  |
| pY10 | pYEXTHS-BN::*Blap*FAR-J |  |
| pY11 | pYEXTHS-BN::*Blap*FAR-A4 |  |
| pY12 | pYEXTHS-BN::*Blap*FAR-A5 |  |

**Yeast strains:**

| **Name** | **Genotype** | **Origin** |
| --- | --- | --- |
| NC/empty | BY4741 pYEXTHS-BN | This study |
| Y1 | BY4741 pY1 |  |
| Y2 | BY4741 pY2 |  |
| Y3 | BY4741 pY3 |  |
| Y4 | BY4741 pY4 |  |
| Y5 | BY4741 pY5 |  |
| Y6 | BY4741 pY6 |  |
| Y7 | BY4741 pY7 |  |
| Y8 | BY4741 pY8 |  |
| Y9 | BY4741 pY9 |  |
| Y10 | BY4741 pY10 |  |
| Y11 | BY4741 pY11 |  |
| Y12 | BY4741 pY12 |  |

**References:**

Horňáková, D., Matoušková, P., Kindl, J., Valterová, I., Pichová, I. 2010. Selection of reference genes for real-time polymerase chain reaction analysis in tissues from *Bombus terrestris* and *Bombus lucorum* of different ages. *Anal. Biochem.* **397,** 118–20.
